# A Systematic Review of the Methods Used in Rapid Approaches to Research and Evaluation

**DOI:** 10.1177/0193841X251338536

**Published:** 2025-04-26

**Authors:** Sigrún Eyrúnardóttir Clark, Norha Vera San Juan, Thomas Moniz, Rebecca Appleton, Phoebe Barnett, Cecilia Vindrola-Padros

**Affiliations:** 1 Rapid Research, Evaluation and Appraisal Lab (RREAL), Department of Targeted Intervention, 4919 University College London, London, UK; 2 NIHR Mental Health Policy Research Unit, Division of Psychiatry, 4919 University College London, London, UK; 3 Clinical, Education & Health Psychology, Division of Psychology & Language Sciences, 4919 University College London, London, UK

**Keywords:** Rapid research and evaluation, Methodology, Reporting standards

## Abstract

Rapid approaches are essential when resources are limited and when findings are required in real-time to inform decisions. Limitations exist in their design and implementation, which can lead to a reduced level of trust in findings. This review sought to map the methods used across rapid evaluations and research to facilitate timeliness and support the rigour of studies. Four scientific databases and one search engine were searched between 11–16th August 2022. Screening led to the inclusion of 169 articles that provided a much-needed repository of methods that can be used during the design and implementation of rapid studies to improve their trustworthiness. No reporting guidelines specific to rapid research or evaluation were identified in the literature, we therefore suggest that this repository of methods informs the development of transparent reporting standards for future rapid research and evaluation.

## Introduction

There is often a need to conduct evaluations and research rapidly to deliver findings in a timely way so these can be used to inform decision-making processes (Beebe, 1995; Johnson & Vindrola-Padros, 2017; McNall & Foster-Fishman, 2007). While prolonged in-depth approaches are most appropriate in some occasions, rapid approaches are vital in contexts where time and resources are limited such as humanitarian crises or complex health emergencies, and real-time evaluation of changing programmes and services (Anker et al., 1993; Beebe, 1995; Gawaya et al., 2022; Holdsworth et al., 2020; Johnson & Vindrola-Padros, 2017; McNall & Foster-Fishman, 2007).

There is a wealth of research on the different types of studies that can be classified as rapid approaches (McNall & Foster-Fishman, 2007; Norman et al., 2022; Vindrola-Padros, 2021; Vindrola-Padros et al., 2020). Rapid research commonly refers to findings that are delivered in the context of time and resource constraints, and rapid evaluation commonly refers to the delivery of timely evidence to inform decision-making and service delivery (Vindrola-Padros, 2021). Concerns have been raised previously that the rapid approach to research and evaluation can be seen as a ‘quick and dirty’ exercise that may impact the quality of data being collected and analysed (Pink & Morgan, 2013). Similarly there have been concerns around the transparency of such studies, with many studies failing to report on the methods used (Vindrola-Padros & Vindrola-Padros, 2018).

Lincoln and Guba's evaluative criteria has been used to assess qualitative research studies to establish trustworthiness in research findings (Enworo, 2023; Forero et al., 2018; Lincoln & Guba, 1985). The criteria considers four key domains in order for research to be considered trustworthy, it should harness approaches that establish credibility, transferability, dependability, and confirmability (Lincoln & Guba, 1985). Authors have previously conducted reviews to identify some of the approaches used in rapid studies, and many of the cited approaches can fit within Lincoln and Guba’s criteria (Beebe, 1995; Fitch et al., 2004; Johnson & Vindrola-Padros, 2017; McNall & Foster-Fishman, 2007; Norman et al., 2022; Vindrola-Padros & Vindrola-Padros, 2018). Beebe’s work focussed on the techniques used in rapid appraisals based on literature in and prior to 1995; Fitch et al. reviewed the techniques used in rapid assessments in the substance use field in 2004; McNall and Foster-Fishman identified the approaches used in rapid evaluation, assessment and appraisal research based on literature in and prior to 2007. These reviews identified key features shared across all rapid evaluation and appraisal methods such as community participation, systems perspective, triangulation using different methods and sources of data, and iterative processes for data collection and analysis. However, all three reviews were published before 2007, limiting our understanding of new developments in this field.

There have been more recent reviews on rapid approaches by Johnson and Vindrola-Padros (reviewed the rapid qualitative methods used during complex health emergencies) (Johnson & Vindrola-Padros, 2017); Vindrola-Padros and Vindrola-Padros (reviewed the approaches used in rapid ethnographies in healthcare organisations) (Vindrola-Padros & Vindrola-Padros, 2018); and Norman et al. (reviewed the approaches used across rapid evaluation studies in healthcare in high-income countries) (Norman et al., 2022). These reviews highlighted the aspects of rapid research and evaluation that might come under scrutiny due to time pressures, such as sampling procedures, approaches for ethical approval, maintaining consistency across members of the research team, and little time to cross-check data with other sources to reduce bias. However, these reviews included publications that were limited to either complex health emergencies or healthcare. We plan to create an updated review to include publications that focus on both health emergencies, healthcare and any other contexts, as a means to create a larger repository of approaches used across a broader context of rapid research and evaluation.

The purpose of this systematic review was to build on this existing literature to produce an updated analysis of the rapid approaches that have been used across multiple sectors; the main challenges faced by researchers and evaluators; and the strategies used to overcome these challenges. The research questions guiding this review were:

• What approaches have been used during rapid evaluation and rapid research to facilitate rigour and timeliness?
• What are the main barriers experienced within these studies, and have any strategies been used to address them?

## Methods

The systematic review was guided by the Preferred Reporting Items for Systematic Reviews and Meta-Analyses (PRISMA) 2020 statement (Page et al., 2021). A protocol outlining the project steps was accepted by PROSPERO prior to conducting the research in August 2022 (CRD42022341825).

### Search Strategy

The search strategy for the review included both keyword and subject heading searches across four scientific literature databases: MEDLINE, Embase, Healthcare Management Information Consortium (HMIC), Cumulative Index of Nursing and Allied Health Literature (CINAHL) Plus, and the open search engine Google Scholar. Search words were related to ‘rapid evaluation’ or ‘rapid research’. The searches were conducted between 11–16th August 2022. A detailed outline of the search strategy can be found in Online Appendix 1.

### Eligibility Criteria

Criteria for including studies consisted of (1) study referred to as a rapid approach; (2) the study having been completed within 6 months (in keeping with rapid approaches) (Vindrola-Padros, 2021); and (3) the article including sufficient information on the methods used to ensure rapidity and support rigour. We define rigour as using approaches that may enhance the trustworthiness of the research (Lincoln & Guba, 1985). There were no restrictions on publication date, language or study design in terms of being qualitative, quantitative or mixed methods.

### Selection Process

The search results were imported into EndNote for de-duplication (Gotschall, 2021), and then to Rayyan for further de-duplication and screening (Ouzzani et al., 2016). The title and abstract screening based on the eligibility criteria was split between three researchers due to the large number of search results. The three researchers then cross-checked 10% of each other’s exclusions and discussed any disagreements of exclusion decisions until a consensus on the decision was reached. Following this, one researcher progressed the included articles to full text screening against the eligibility criteria, using Microsoft Excel to report the screening decision. Any articles identified in non-English languages were translated using Google Translate. The principal investigator then cross-checked 10% of the excluded articles.

**Figure 1.**
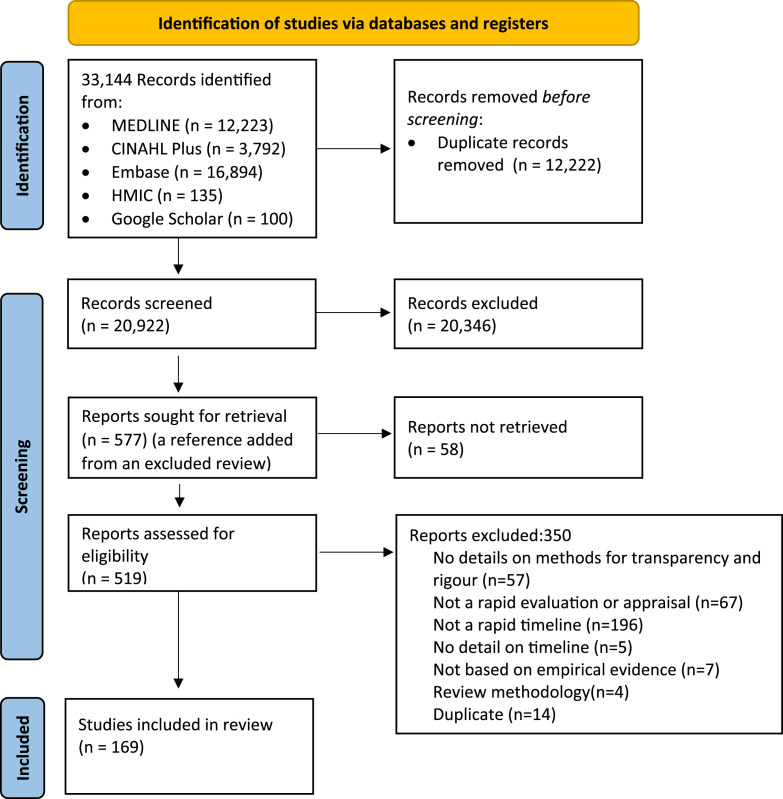
Prisma 2020 flow diagram of the selection process.

The search returned 33,144 results, of which 20,922 articles remained following duplicate removal. Of these, 577 articles were identified as potentially relevant and screened at full text. Overall, 169 articles were included in the review. Figure 1 shows the flow of studies through the review process based on PRISMA guidelines (Page et al., 2021).

The reasons for excluding articles included: articles not sharing enough detail on the methods used to ensure rapidity and rigour; the study not fitting under a variation of rapid evaluation or research; the study not being conducted to a rapid timeline (within 6 months); no detail on the study timeline; the study not being based on empirical evidence; the study using review methodology; or the study was a duplicate of a previously screened item.

### Data Extraction and Quality Assessment

A comprehensive list of data extraction categories was developed based on the research question and pre-specified outcomes of interest. These categories were further refined to ensure information was captured appropriately following screening. Data extraction, quality assessments and full text screening were carried out in parallel by one researcher, recording information on Microsoft Excel. The final data extraction categories included: study details (location of study; study design; study duration; general methods used throughout) and the methods used across the different stages of the rapid studies (as shown in Online Appendix 2) such as during study design; data collection; data analysis; result interpretation; and dissemination. We also extracted any method limitations outlined in the articles.

The Mixed Methods Appraisal Tool (MMAT) (Hong et al., 2018) was used to assess the quality of the included articles. The MMAT allows the quality of heterogeneous study designs to be assessed based on the methodology used. The types of study that can be assessed with this tool include qualitative, quantitative randomised control, quantitative non-randomised control, quantitative descriptive, and mixed methods studies. No articles were excluded based on their MMAT scores.

The MMAT scores of each study can be found in Online Appendix 2. The studies ranged from low quality with a score of 0/5 (*n* = 3) and 1/5 (*n* = 32) to medium quality with a score of 2/5 (*n* = 38) and 3/5 (*n* = 25), to high quality with a score of 4/5 (*n* = 61) and 5/5 (*n* = 10).

### Data Synthesis

Textual narrative synthesis and a summary of content were conducted to summarise the study characteristics and key findings across the literature (Lucas et al., 2007). We then used the rigorous and accelerated data reduction technique (RADaR) to reduce the data within our findings (Watkins, 2017). The RADaR technique consists of using a sequence of tables to chart findings with the aim of reducing the volume of data with each subsequent table.

## Results

### Study Characteristics

A detailed summary of the characteristics and themes of all the studies included in the review can be found in Online Appendix 2, and the full list of included references can be found in Online Appendix 3.

Most of the included studies were conducted in the USA (*n* = 35), followed by the UK (*n* = 12), South Africa (*n* = 9), Uganda (*n* = 8), and Kenya (*n* = 7), more details on study location can be found in Online Appendix 2. Rapid assessments were the most common form of rapid study based on reports from authors on the study designs they had used, followed by rapid appraisals and rapid evaluations, more detail can be found in Table 1 below. A large majority of the rapid approaches were conducted within qualitative study designs.

**Table 1.** Summary of the Study Designs of Included Publications.

Characteristics	Number of studies
Type of study design	
Qualitative	110
Quantitative	21
Mixed methods	38
Type of rapid study	
Rapid evaluation	13
Rapid appraisal	31
Rapid rural appraisal	3
Participatory rapid appraisal	3
Participatory rural appraisal	9
Participatory needs assessment	1
Rapid assessment	88
Rapid assessment response	6
Rapid assessment response and evaluation	2
Rapid rural assessment	1
Peer ethnography	1
Rapid assessment procedure informed clinical ethnography	1
Rapid ethnographic assessment	2
Rapid ethnographic evaluation	1
Participatory evaluation	1
Evaluation	4
Rapid analysis	2

### Methods, Challenges and Mitigation Strategies Reported Across the Rapid Studies 

Summarised below are the methods used across the included publications that enabled rapidity and supported rigour, along with some of the challenges associated with these methods and any mitigation strategies as reported by the literature. A detailed summary of the methods used across the included literature can be found in Online Appendix 2.

#### Reporting Guidelines

A prominent gap across the literature was that only four studies discussed the use of specific guidelines for the conduct and reporting of the studies, and all four used the qualitative research (CORE-Q) guidelines.

Theoretical frameworks and approaches were cited more commonly (*n* = 30 studies) to guide study conduct, the most commonly cited were the Consolidated Framework for Implementation Research (*n* = 3), the Health Belief Model (*n* = 3), the International Rapid Assessment Response and Evaluation (I-RARE)/RARE methodology (*n* = 4), and the Rapid Assessment Procedure-Informed Clinical Ethnography (RAPICE) methodology (*n* = 3).

#### Study Design

A key area discussed among the publications was the use of sampling methodologies to increase rapidity or to improve relevance of characteristics of the sample such as non-probability sampling (used across 84 studies) in the form of convenience sampling, purposive sampling or variations of snowball sampling. Some studies (*n* = 14) based their sample size on when data saturation was reached. Small sample sizes and relying on non-probability sampling was recognised as a limitation across some publications, as it led to limited generalisability, limited representativeness of findings and the potential to introduce selection bias (Ahoua et al., 2006; Akello et al., 2007; Anastasaki et al., 2022; Brittain et al., 2019; Butler et al., 2021; Ezard et al., 2011; Hammond et al., 2022; Johnson & Vindrola-Padros, 2017; Morojele et al., 2006; Negandhi et al., 2017; Nemser et al., 2018; Sy et al., 2020; Theiss-Nyland et al., 2016). Three articles however used methods to minimise these limitations by searching for alternative or disconfirming cases, deviating from arranged locations to try to reach unsensitized groups, or using active, sequential recruitment to reduce the risk of excluding participants with underrepresented characteristics (Grant et al., 2011; Kahle et al., 2009; Kamineni et al., 2011).

Other approaches used throughout the study design of the included publications included following a study protocol/proposal, gaining relevant ethical and regulatory approvals, obtaining informed consent from participants ahead of data collection in the form of verbal, written and assumed consent (based on voluntary participation in online surveys and observations). Approaches to recruitment were also reported, where some studies (*n* = 22) were supported by local key informants, local researchers/evaluators, local networks, and organisations to build trust with potential study participants and to advertise the study. Other studies (*n* = 16) relied on emails or social media platforms to recruit participants. Some studies incentivised participants to improve recruitment through monetary incentives (*n* = 23), food and drink incentives (*n* = 6), and health incentives (*n* = 4).

#### Data Collection and Analysis

Methods of data collection and analysis that enabled rapidity included relying on team members to split data collection and analysis between each other. However inconsistency in data collection methods and data analysis methods, due to not having the time to train team members or having the time for supervisors to attend all data collection to ensure alignment across team members, were identified as challenges in the rapid studies (Ash et al., 2016; Bayleyegn et al., 2006). Methods such as team meetings during data collection and analysis were used frequently to address this challenge (*n* = 42) as a way for team members to share feedback with each other to ensure consistency with the methods used, achieve consensus on approaches used, and to modify any processes or materials (Abir et al., 2021; Albert et al., 2021; Sy et al., 2020).

Piloting data collection tools was conducted across 26 studies to support their rigour and in some of the studies this was done to ensure cultural and face validity. Several authors however reflected on the fact that short timelines often limited the piloting of data collection instruments and inhibited team members from going in depth to ask certain questions to immerse themselves into data collection (Ezard et al., 2011; Seidel et al., 2018; Tindana et al., 2012). Strategies to overcome these challenges included using member checking (cited across 27 studies) to share preliminary findings with the participants themselves or with members of their community, as a way to corroborate the analysis and interpretations that had been conducted (Belford et al., 2017; Chilanga et al., 2022; Chung et al., 2022). Additionally, triangulating findings with other methods of data collection or existing documents was often cited across the literature (*n* = 82) as a way to verify findings and interpretations (Balogh et al., 2008; Doherty et al., 2017; Loko et al., 2019).

Iterative approaches to data collection and data analysis were used across 32 studies whereby the studies analysed data whilst data collection was ongoing allowing teams to identify when data saturation had been met, to re-shape data collection tools based on emerging findings and to develop initial codebooks for subsequent in-depth analysis (Albert et al., 2021; Aral et al., 2005; Jumbe et al., 2021). Many of the studies (*n* = 66) conducted data collection and analysis in local languages working with translators, local researchers, and with researchers with lived experience. There were nine studies that discussed how this helped to facilitate trust and strong validity of the cultural and traditional knowledge (Anastasaki et al., 2022; Brown et al., 2008; Hanvoravongchai et al., 2010).

Other approaches used during the data collection and analysis included: reporting the composition and experience of the evaluation or research team (*n* = 87), using participatory methods for data collection (*n* = 14), relying on technology as a medium to collect data from participants (23), and recording and transcribing interviews or using field notes (*n* = 84). Many studies detailed their analysis approaches predominantly qualitative content analysis (*n* = 65) such as thematic analysis and the framework approach, also discussed were rapid analysis techniques such as the use of rapid assessment procedure sheets (*n* = 11). Some studies (*n* = 23) also used quality assurance approaches and audit trails to assess consistency in analysis across team members and to assess the quality of the analysed data.

#### Result Interpretation

Limitations can exist when team members do not recognise the effect their characteristics and experiences may have on their interpretations of findings. However a few studies shared that their team were actually able to reflect on their practice throughout the study and how their personal experiences may have affected their interpretations (Dasgupta et al., 2008; Laisser et al., 2021; Mital et al., 2016). Some studies (*n* = 10) had a separate peer-review team or advisory group that reviewed the research/evaluation teams’ interpretations and the draft reports to identify any potential biases.

#### Dissemination

Iterative dissemination of findings whilst studies were ongoing was used in three studies to rapidly share emerging findings with study participants, research team members, commissioners, and implementers of programs being evaluated (Burgess-Allen & Owen-Smith, 2010; Cohn et al., 2021; Vindrola-Padros et al., 2020). This formed feedback loops between stakeholders implementing findings and the evaluation/research team, enabling evaluation of studies or programs as they are being implemented.

## Discussion

The aim of this review was to identify the methods that have been used by evaluators and researchers to carry out studies in the context of time pressures. The review is a response to current debates in the literature on the rigour and transparency of rapid studies, where short timeframes are often associated with ‘quick and dirty’ exercises, and limited reporting of the study methodology (Cupit et al., 2018; Vindrola-Padros, 2021; Vindrola-Padros & Vindrola-Padros, 2018). The findings from our review demonstrate that many of the rapid studies use approaches to improve their rigour, opposing the opinion that rapid approaches are ‘quick and dirty’. Many of the approaches identified in this review could be grouped into the areas of Lincoln and Guba’s criteria to facilitate trustworthiness of research findings (Lincoln & Guba, 1985). Approaches ensuring credibility included triangulation and member checking; establishing transferability had been facilitated by relying on local key informants and local researchers to support with understanding the local cultural and social contexts; establishing dependability had been achieved through auditing and peer-reviewing the research or evaluation; and establishing confirmability took place through approaches of reflexivity.

We found similarities in our review with the previous literature in terms of the approaches used by researchers and evaluators. This included relying on approaches to facilitate timely recruitment such as non-probability sampling (Beebe, 1995; Norman et al., 2022; Vindrola-Padros & Vindrola-Padros, 2018) and using existing networks to support with recruitment (Vindrola-Padros & Vindrola-Padros, 2018). Approaches to rapidly collect and analyse data were also discussed such as team-based approaches for data collection and analysis (Beebe, 1995); technology to support with data collection (Norman et al., 2022); rapid analysis techniques (Norman et al., 2022; Rankl et al., 2021); and an iterative process of data collection, analysis, and dissemination (Beebe, 1995; Norman et al., 2022; Rankl et al., 2021).

A key finding from our review that has not been highlighted in the previous reviews, was that very few publications cited the use of guidelines to enable rigour within the conduct and reporting of studies. The CORE-Q checklist (Tong et al.,. 2007) was the only reported guideline in our systematic review, which primarily shares guidance on research team dynamics, study design, data collection, analysis, and synthesis in qualitative research only, and does not specifically consider the design of rapid studies. We therefore recognise this as a gap in the field where reporting guidelines for rapid research and evaluation would be helpful. The approaches summarised in this review and previous reviews, could serve as a starting point of components that would be useful for inclusion in reporting guidelines for rapid studies. Further work is needed to generate consensus among rapid researchers and evaluators regarding the components that need to be included in guidelines for rapid evaluation and research. Another finding from our review that links to previous literature (Vindrola-Padros & Vindrola-Padros, 2018) is that some rapid studies are still failing to transparently report on the methods and approaches used, this was recognised in our review when 57 rapid studies (14% of the full text exclusions) were excluded at full text screening because of failing to report on methodologies used. We hope future rapid researchers and evaluators will consider this and transparently share the methods used when designing, implementing and reporting on rapid studies.

The strengths of this review include the wealth of incorporated studies (*n* = 169) that have been published over a relatively long period of time (1993–2022). One of the aims of this review was to create a repository of approaches used across studies published at a global scale, but we found that there is an overwhelming majority of publications from high-income countries (USA (*n* = 35) and UK (*n* = 12)). This represents a limitation regarding the transferability of these review findings to the context of Low and Middle Income Countries (LMICs) and highlights an area that future research should address by delivering and publishing rapid studies set in LMICs. Other limitations include the fact the review focused on articles published in peer-reviewed journals, excluding rapid evaluations and research in the grey literature; and that study durations of six months or less was used as a component of the inclusion criteria in our review, so articles that did not report study duration had to be excluded.

## Conclusion

This systematic review has collated evidence that goes against the opinion that rapid studies are ‘quick and dirty’, as approaches are being used in rapid studies that fit within Lincoln and Guba’s framework for ensuring trustworthiness of research (Lincoln & Guba, 1985). A gap in this field has been identified that no reporting guidelines for rapid studies exist or are being used. The development of reporting guidelines could ensure rapid studies continue to be designed and delivered to produce trustworthy findings, and could help to address the finding from our review that some rapid studies are still failing to clearly report on methods used. Our research team plans to develop reporting standards based on the approaches identified in this review and with consultation of key stakeholders in the field, to facilitate the future transparent reporting and uptake of methods that enable speed while maintaining the rigour of rapid evaluation and research.

## Supplemental Material

Supplemental Material - A systematic review of the methods used in rapid approaches to research and evaluation Supplemental Material for A systematic review of the methods used in rapid approaches to research and evaluation by Sigrún Eyrúnardóttir Clark, Norha Vera San Juan, Thomas Moniz, Rebecca Appleton, Phoebe Barnett and Cecilia Vindrola-Padros in Evaluation Review.

## References

[bibr1-0193841X251338536] Abir M. Forman J. Taymour R. K. Brent C. Nallamothu B. K. Scott J. Wahl K. (2021). Optimizing routine and disaster prehospital care through improved emergency medical services oversight. Disaster Medicine and Public Health Preparedness, 15(5), 595–607. 10.1017/dmp.2020.71 32476635

[bibr2-0193841X251338536] Ahoua L. Tamrat A. Duroch F. Grais R. F. Brown V. (2006). High mortality in an internally displaced population in ituri, democratic republic of Congo, 2005: Results of a rapid assessment under difficult conditions. Global Public Health, 1(3), 195–204. 10.1080/17441690600681869 19153907

[bibr3-0193841X251338536] Akello G. Reis R. Ovuga E. Rwabukwali C. B. Kabonesa C. Richters A. (2007). Primary school children’s perspectives on common diseases and medicines used: Implications for school healthcare programmes and priority setting in Uganda. African Health Sciences, 7(2), 73–79. 10.5555/afhs.2007.7.2.73 17594283 PMC1925265

[bibr4-0193841X251338536] Albert S. L. Paul M. M. Nguyen A. M. Shelley D. R. Berry C. A. (2021). A qualitative study of high-performing primary care practices during the COVID-19 pandemic. BMC Family Practice, 22(1), 237. 10.1186/s12875-021-01589-4 34823495 PMC8614080

[bibr5-0193841X251338536] Anastasaki M. van Bree E. M. Brakema E. A. Tsiligianni I. Sifaki-Pistolla D. Chatzea V. E. Crone M. C. Karelis A. van der Kleij R. M. J. J. Poot C. C. Reis R. Chavannes N. H. Lionis C. (2022). Beliefs, perceptions, and behaviors regarding chronic respiratory diseases of roma in crete, Greece: A qualitative fresh air study. Frontiers in Public Health, 10, Article 812700. 10.3389/fpubh.2022.812700 PMC9051233 35493388

[bibr6-0193841X251338536] Anker M. Guidotti R. J. Orzeszyna S. Sapirie S. A. Thuriaux M. C. (1993). Rapid evaluation methods (REM) of health services performance: Methodological observations. Bulletin of the World Health Organization, 71(1), 15–21. 8440033 PMC2393426

[bibr7-0193841X251338536] Aral S. O. St Lawrence J. S. Dyatlov R. Kozlov A. (2005). Commercial sex work, drug use, and sexually transmitted infections in St. Petersburg, Russia. Social Science & Medicine, 60(10), 2181–2190. 10.1016/j.socscimed.2004.10.009 15748667

[bibr8-0193841X251338536] Ash J. S. Chase D. Wiesen J. F. Murphy E. V. Marovich S. (2016). Studying readiness for clinical decision support for worker health using the rapid assessment process and mixed methods interviews. AMIA. Annual Symposium Proceedings. AMIA Symposium, 2016, 285–294. 28269822 PMC5333245

[bibr9-0193841X251338536] Balogh R. Whitelaw S. Thompson J. (2008). Rapid needs appraisal in the modern NHS: Potential and dilemmas. Critical Public Health, 18(2), 233–244. 10.1080/09581590701377010

[bibr10-0193841X251338536] Bayleyegn T. Wolkin A. Oberst K. Young S. Sanchez C. Phelps A. Schulte J. Rubin C. Batts D. (2006). Rapid assessment of the needs and health status in santa rosa and escambia counties, Florida, after hurricane ivan, september 2004. Disaster Management & Response: DMR: an official publication of the Emergency Nurses Association, 4(1), 12–18. 10.1016/j.dmr.2005.10.001 16360635

[bibr11-0193841X251338536] Beebe J. (1995). Basic concepts and techniques of rapid appraisal. Human Organization, 54(1), 42–51. 10.17730/humo.54.1.k84tv883mr2756l3

[bibr12-0193841X251338536] Belford M. Robertson T. Jepson R. (2017). Using evaluability assessment to assess local community development health programmes: A scottish case-study. BMC Medical Research Methodology, 17(1), 70. 10.1186/s12874-017-0334-4 28431505 PMC5399800

[bibr13-0193841X251338536] Brittain A. W. August E. M. Romero L. Sheahan M. Krashin J. Ntansah C. Honein M. A. Jamieson D. J. Ellis E. M. Davis M. S. Lathrop E. (2019). Community perspectives on contraception in the context of the zika virus in the U.S. Virgin islands: Implications for communication and messaging. Women's Health Issues: Official Publication of the Jacobs Institute of Women's Health, 29(3), 245–251. 10.1016/j.whi.2019.01.007 PMC6707511 30878263

[bibr14-0193841X251338536] Brown D. R. Hernández A. Saint-Jean G. Evans S. Tafari I. Brewster L. G. Celestin M. J. Gómez-Estefan C. Regalado F. Akal S. Nierenberg B. Kauschinger E. D. Schwartz R. Page J. B. (2008). A participatory action research pilot study of urban health disparities using rapid assessment response and evaluation. American Journal of Public Health, 98(1), 28–38. 10.2105/AJPH.2006.091363 18048802 PMC2156052

[bibr15-0193841X251338536] Burgess-Allen J. Owen-Smith V. (2010). Using mind mapping techniques for rapid qualitative data analysis in public participation processes. Health Expectations: An International Journal of Public Participation in Health Care and Health Policy, 13(4), 406–415. 10.1111/j.1369-7625.2010.00594.x 20550595 PMC5060552

[bibr16-0193841X251338536] Butler J. R. A. Davila F. Alders R. Bourke R. M. Crimp S. McCarthy J. McWilliam A. Palo A. S. M. Robins L. Webb M. J. van Wensveen M. Sanderson T. Walker D. (2021). A rapid assessment framework for food system shocks: Lessons learned from COVID-19 in the Indo-Pacific region. Environmental Science & Policy, 117, 34–45. 10.1016/j.envsci.2020.12.011 34744509 PMC8556181

[bibr17-0193841X251338536] Chilanga E. Dzimbiri M. Mwanjawala P. Keller A. Mbeya R. A. (2022). Religion, politics and COVID-19 risk perception among urban residents in Malawi. BMC Public Health, 22(1), 1430. 10.1186/s12889-022-13858-7 35897087 PMC9326149

[bibr18-0193841X251338536] Chung A. D. Kwan B. Y. M. Wagner N. Braund H. Hanmore T. Hall A. K. McEwan L. Dalgarno N. Dagnone J. D. (2022). An adaptation-focused evaluation of Canada’s first competency-based medical education implementation in radiology. European Journal of Radiology, 147, Article 110109. 10.1016/j.ejrad.2021.110109 34968900

[bibr19-0193841X251338536] Cohn W. F. Canan C. E. Knight S. Waldman A. L. Dillingham R. Ingersoll K. Schexnayder J. Flickinger T. E. (2021). An implementation strategy to expand mobile health use in HIV care settings: Rapid evaluation study using the consolidated framework for implementation research. JMIR MHealth and UHealth, 9(4), Article e19163. 10.2196/19163 33908893 PMC8116995

[bibr20-0193841X251338536] Cupit C. Mackintosh N. Armstrong N. (2018). Using ethnography to study improving healthcare: Reflections on the ‘ethnographic’ label. BMJ Quality and Safety, 27(4), 258–260. 10.1136/bmjqs-2017-007599 29463770

[bibr21-0193841X251338536] Dasgupta R. Chaturvedi S. Adhish S. V. Ganguly K. K. Rai S. Sushant L. Arora N. K. (2008). Social determinants and polio ‘endgame’: A qualitative study in high risk districts of India. Indian Pediatrics, 45(5), 357–365. 18693373

[bibr22-0193841X251338536] Doherty T. Besada D. Goga A. Daviaud E. Rohde S. Raphaely N. (2017). ‘If donors woke up tomorrow and said we can’t fund you, what would we do?’ A health system dynamics analysis of implementation of PMTCT option B+ in Uganda. Globalization and Health, 13(1), 51. 10.1186/s12992-017-0272-2 28747196 PMC5530517

[bibr23-0193841X251338536] Enworo O. C. (2023). Application of Guba and Lincoln's parallel criteria to assess trustworthiness of qualitative research on indigenous social protection systems. Qualitative Research Journal, 23(4), 372–384. 10.1108/QRJ-08-2022-0116

[bibr24-0193841X251338536] Ezard N. Oppenheimer E. Burton A. Schilperoord M. Macdonald D. Adelekan M. Sakarati A. van Ommeren M. (2011). Six rapid assessments of alcohol and other substance use in populations displaced by conflict. Conflict and Health, 5(1), 1. 10.1186/1752-1505-5-1 21310092 PMC3050731

[bibr25-0193841X251338536] Fitch C. Stimson G. V. Rhodes T. Poznyak V. (2004). Rapid assessment: An international review of diffusion, practice and outcomes in the substance use field. Social Science & Medicine, 59(9), 1819–1830. 10.1016/j.socscimed.2004.02.028 15312917

[bibr26-0193841X251338536] Forero R. Nahidi S. De Costa J. Mohsin M. Fitzgerald G. Gibson N. McCarthy S. Aboagye-Sarfo P. (2018). Application of four-dimension criteria to assess rigour of qualitative research in emergency medicine. BMC Health Services Research, 18(1), 120. 10.1186/s12913-018-2915-2 29454350 PMC5816375

[bibr27-0193841X251338536] Gawaya M. Terrill D. Williams E. (2022). Using rapid evaluation methods to assess service delivery changes: Lessons learned for evaluation practice during the COVID-19 pandemic. Evaluation Journal of Australasia, 22(1), 30–48. 10.1177/1035719X211057630 35261532 PMC8891248

[bibr28-0193841X251338536] Gotschall T. (2021). EndNote 20 desktop version. Journal of the Medical Library Association: JMLA, 109(3), 520–522. 10.5195/jmla.2021.1260 34629985 PMC8485940

[bibr29-0193841X251338536] Grant L. Brown J. Leng M. Bettega N. Murray S. A. (2011). Palliative care making a difference in rural Uganda, Kenya and Malawi: Three rapid evaluation field studies. BMC Palliative Care, 10, 8. 10.1186/1472-684X-10-8 21569423 PMC3120792

[bibr30-0193841X251338536] Hammond N. Steels S. King G. (2022). Contraceptive and pregnancy concerns in the UK during the first COVID-19 lockdown: A rapid study. Sexual & reproductive healthcare: Official Journal of the Swedish Association of Midwives, 33, Article 100754. 10.1016/j.srhc.2022.100754 PMC9270775 35842979

[bibr31-0193841X251338536] Hanvoravongchai P. Adisasmito W. Chau P. N. Conseil A. de Sa J. Krumkamp R. Mounier-Jack S. Phommasack B. Putthasri W. Shih C.-S. Touch S. Coker R. AsiaFluCap Project . (2010). Pandemic influenza preparedness and health systems challenges in asia: Results from rapid analyses in 6 asian countries. BMC Public Health, 10, 322. 10.1186/1471-2458-10-322 20529345 PMC2896940

[bibr32-0193841X251338536] Holdsworth L. M. Safaeinili N. Winget M. Lorenz K. A. Lough M. Asch S. Malcolm E. (2020). Adapting rapid assessment procedures for implementation research using a team-based approach to analysis: A case example of patient quality and safety interventions in the ICU. Implementation Science: Iscus, 15(1), 12. 10.1186/s13012-020-0972-5 PMC7036173 32087724

[bibr33-0193841X251338536] Hong Q. N. Fàbregues S. Bartlett G. Boardman F. Cargo M. Dagenais P. Gagnon M.-P. Griffiths F. Nicolau B. O’Cathain A. Rousseau M.-C. Vedel I. Pluye P. (2018). The Mixed Methods Appraisal Tool (MMAT) version 2018 for information professionals and researchers. Education for Information, 34(4), 285–291. 10.3233/EFI-180221

[bibr34-0193841X251338536] Johnson G. A. Vindrola-Padros C. (2017). Rapid qualitative research methods during complex health emergencies: A systematic review of the literature. Social Science & Medicine, 189, 63–75. 10.1016/j.socscimed.2017.07.029 28787628

[bibr35-0193841X251338536] Jumbe S. Milner A. Clinch M. Kennedy J. Pinder R. J. Sharpe C. A. Fenton K. (2021). A qualitative evaluation of Southwark Council’s public health response to mitigating the mental health impact of the 2017 London bridge and borough market terror attack. BMC Public Health, 21(1), 1427. 10.1186/s12889-021-11447-8 34281513 PMC8290577

[bibr36-0193841X251338536] Kahle E. M. Barash E. A. Page L. C. Lansky A. Jafa K. Sullivan P. S. Buskin S. E. (2009). Evaluation of the impact of news coverage of an HIV multiclass drug-resistant cluster in Seattle, Washington. American Journal of Public Health, 99(Suppl 1), S131–S136. 10.2105/AJPH.2007.126656 19218180 PMC2724959

[bibr37-0193841X251338536] Kamineni V. V. Turk T. Wilson N. Satyanarayana S. Chauhan L. S. (2011). A rapid assessment and response approach to review and enhance advocacy, communication and social mobilisation for tuberculosis control in Odisha state, India. BMC Public Health, 11, 463. 10.1186/1471-2458-11-463 21663623 PMC3141449

[bibr38-0193841X251338536] Laisser R. Danna V. A. Bonet M. Oladapo O. T. Lavender T. (2021). An exploration of midwives’ views of the latest World Health Organization labour care guide. African Journal of Midwifery and Women's Health, 15(4), 1–11. 10.12968/ajmw.2020.0043

[bibr39-0193841X251338536] Lincoln Y. S. Guba E. G. (1985). Naturalistic inquiry. Newberry Park.

[bibr40-0193841X251338536] Loko L. E. Y. Medegan Fagla S. Orobiyi A. Glinma B. Toffa J. Koukoui O. Djogbenou L. Gbaguidi F. (2019). Traditional knowledge of invertebrates used for medicine and magical–religious purposes by traditional healers and indigenous populations in the Plateau Department, Republic of Benin. Journal of Ethnobiology and Ethnomedicine, 15(1), 66. 10.1186/s13002-019-0344-x 31842934 PMC6916055

[bibr41-0193841X251338536] Lucas P. J. Baird J. Arai L. Law C. Roberts H. M. (2007). Worked examples of alternative methods for the synthesis of qualitative and quantitative research in systematic reviews. BMC Medical Research Methodology, 7, 4. 10.1186/1471-2288-7-4 17224044 PMC1783856

[bibr42-0193841X251338536] McNall M. Foster-Fishman P. G. (2007). Methods of rapid evaluation, assessment, and appraisal. American Journal of Evaluation, 28(2), 151–168. 10.1177/1098214007300895

[bibr43-0193841X251338536] Mital S. Miles G. McLellan-Lemal E. Muthui M. Needle R. (2016). Heroin shortage in coastal Kenya: A rapid assessment and qualitative analysis of heroin users’ experiences. International Journal of Drug Policy, 30, 91–98. 10.1016/j.drugpo.2015.08.010 26470646 PMC4762754

[bibr44-0193841X251338536] Morojele N. K. Kachieng’a M. A. Mokoko E. Nkoko M. A. Parry C. D. H. Nkowane A. M. Moshia K. M. Saxena S. (2006). Alcohol use and sexual behaviour among risky drinkers and bar and shebeen patrons in Gauteng province, South Africa. Social Science & Medicine, 62(1), 217–227. 10.1016/j.socscimed.2005.05.031 16054281

[bibr45-0193841X251338536] Negandhi H. Tiwari R. Sharma A. Nair R. Zodpey S. Reddy Allam R. Oruganti G. (2017). Rapid assessment of facilitators and barriers related to the acceptance, challenges and community perception of daily regimen for treating tuberculosis in India. Global Health Action, 10(1), Article 1290315. 10.1080/16549716.2017.1290315 28485693 PMC5496091

[bibr46-0193841X251338536] Nemser B. Aung K. Mushamba M. Chirwa S. Sera D. Chikhwaza O. Kachale F. (2018). Data-informed decision-making for life-saving commodities investments in Malawi: A qualitative case study. Malawi Medical Journal: The Journal of Medical Association of Malawi, 30(2), 111–119. 10.4314/mmj.v30i2.11 30627339 PMC6307067

[bibr47-0193841X251338536] Norman G. Mason T. Dumville J. C. Bower P. Wilson P. Cullum N. (2022). Approaches to enabling rapid evaluation of innovations in health and social care: A scoping review of evidence from high-income countries. BMJ Open, 12(12), Article e064345. 10.1136/bmjopen-2022-064345 PMC10580278 36600433

[bibr48-0193841X251338536] Ouzzani M. Hammady H. Fedorowicz Z. Elmagarmid A. (2016). Rayyan—a web and mobile app for systematic reviews. Systematic Reviews, 5(1), 210. 10.1186/s13643-016-0384-4 27919275 PMC5139140

[bibr49-0193841X251338536] Page M. J. McKenzie J. E. Bossuyt P. M. Boutron I. Hoffmann T. C. Mulrow C. D. Shamseer L. Tetzlaff J. M. Akl E. A. Brennan S. E. Chou R. Glanville J. Grimshaw J. M. Hróbjartsson A. Lalu M. M. Li T. Loder E. W. Mayo-Wilson E. McDonald S. Moher D. (2021). The PRISMA 2020 statement: An updated guideline for reporting systematic reviews. BMJ, n71, n71. 10.1136/bmj.n71 PMC8005924 33782057

[bibr50-0193841X251338536] Pink S. Morgan J. (2013). Short‐term ethnography: Intense routes to knowing. Symbolic Interaction, 36(3), 351–361. 10.1002/symb.66

[bibr51-0193841X251338536] Rankl F. Johnson G. A. Vindrola-Padros C. (2021). Examining what we know in relation to how we know it: A team-based reflexivity Model for rapid qualitative health research. Qualitative Health Research, 31(7), 1358–1370. 10.1177/1049732321998062 33745367 PMC8182295

[bibr52-0193841X251338536] Seidel S. Muciimi J. Chang J. Gitari S. Keiser P. Goodman M. L. (2018). Community perceptions of home environments that lead children & youth to the street in semi-rural Kenya. Child Abuse & Neglect, 82, 34–44. 10.1016/j.chiabu.2018.05.011 29852364

[bibr53-0193841X251338536] Sy A. Marriott J. Tannis C. Demment M. McIntosh S. Hadley J. Albert P. Buenconsejo-Lum L. Dye T. (2020). A rapid assessment procedure to develop A non-communicable disease prevention pilot health communications project using E- and M-health communications in pohnpei state, Federated States OF Micronesia. Hawai’i Journal of Health & Social Welfare, 79(6 Suppl 2), 58–63. 32596680 PMC7311940

[bibr54-0193841X251338536] Theiss-Nyland K. Ejersa W. Karema C. Koné D. Koenker H. Cyaka Y. Lynch M. Webster J. Lines J. (2016). Operational challenges to continuous LLIN distribution: A qualitative rapid assessment in four countries. Malaria Journal, 15, 131. 10.1186/s12936-016-1184-y 26931237 PMC4774176

[bibr55-0193841X251338536] Tindana P. Bull S. Amenga-Etego L. de Vries J. Aborigo R. Koram K. Kwiatkowski D. Parker M. (2012). Seeking consent to genetic and genomic research in a rural Ghanaian setting: A qualitative study of the MalariaGEN experience. BMC Medical Ethics, 13, 15. 10.1186/1472-6939-13-15 22747883 PMC3441464

[bibr56-0193841X251338536] Tong A. Sainsbury P. Craig J. (2007). Consolidated criteria for reporting qualitative research (COREQ): A 32-item checklist for interviews and focus groups. International Journal for Quality in Health Care: Journal of the International Society for Quality in Health Care, 19(6), 349–357. 10.1093/intqhc/mzm042 17872937

[bibr58-0193841X251338536] Vindrola-Padros C. (2021). Doing rapid qualitative research. Sage.

[bibr59-0193841X251338536] Vindrola-Padros C. Chisnall G. Cooper S. Dowrick A. Djellouli N. Symmons S. M. Martin S. Singleton G. Vanderslott S. Vera N. Johnson G. A. (2020). Carrying out rapid qualitative research during a pandemic: Emerging lessons from COVID-19. Qualitative Health Research, 30(14), 2192–2204. 10.1177/1049732320951526 32865149 PMC7649912

[bibr60-0193841X251338536] Vindrola-Padros C. Vindrola-Padros B. (2018). Quick and dirty? A systematic review of the use of rapid ethnographies in healthcare organisation and delivery. BMJ Quality and Safety, 27(4), 321–330. 10.1136/bmjqs-2017-007226 29263139

[bibr61-0193841X251338536] Watkins D. C. (2017). Rapid and rigorous qualitative data analysis. International Journal of Qualitative Methods, 16(1), Article 160940691771213. 10.1177/1609406917712131

